# Infiltrative Type I Collagen in the Treatment of Morton’s Neuroma: A Mini-Series

**DOI:** 10.3390/jcm12144640

**Published:** 2023-07-12

**Authors:** Federico Giarda, Adele Agostini, Stefano Colonna, Luciana Sciumè, Alberto Meroni, Giovanna Beretta, Davide Dalla Costa

**Affiliations:** 1 Unit of Rehabilitation Medicine and Neurorehabilitation, Department of Neuroscience, ASST Niguarda Hospital, 20162 Milan, Italy; stefano.colonna@ospedaleniguarda.it (S.C.); luciana.sciume@ospedaleniguarda.it (L.S.); davide.dallacosta@ospedaleniguarda.it (D.D.C.); 2 Department of Biomedical Sciences for Health, Università degli Studi di Milano, 20133 Milan, Italy; adeleagostini94@gmail.com; 3 Department of Orthopedic Surgery and Traumatology, ASST Niguarda Hospital, 20162 Milan, Italy; albertomarco.meroni@ospedaleniguarda.it

**Keywords:** Morton’s neuroma, type I porcine collagen, clinical treatment, US-guided injection, case mini-series

## Abstract

Morton’s neuroma (MN) is a compressive neuropathy of the common plantar digital nerve, most commonly affecting the third inter-digital space. The conservative approach is the first recommended treatment option. However, other different approaches have been proposed, offering several options of treatments, where, several degrees of efficacy and safety have been reported. We treated five consecutive patients affected by MN through three indirect ultrasound-guided injections of type I porcine collagen at weekly intervals. All patients were assessed before the treatment, after the treatment and up to 6 months after the last injection via AOFAS and VNS scores for pain, in which the function and pain were evaluated, respectively. In all patients, both analyzed variables progressively ameliorated, with benefits lasting until the last follow-up. The trend of the scores during the follow-up showed significant statistical differences. No side effects occurred. To our knowledge, this is the first study on injections of type I porcine collagen for the treatment of Morton’s neuroma. Future research is needed to confirm the positive trend achieved in this MN mini-series.

## 1. Introduction

Morton’s neuroma (MN) is a compressive neuropathy of the common digital plantar nerve (CDPN). It often causes significant pain that limits footwear choice and weight-bearing activities. This condition was first described anatomically by Civinini in 1835 [1] and later described clinically by Thomas Morton in 1876 [2]. Therefore, some authors have called this pathology Civinini–Morton syndrome (CMS) [3]. Predisposing conditions are female gender; the use of high-heeled, narrow-soled shoes; athletic activities that cause repetitive foot trauma; and foot deformities such as hammertoes, pes cavus or flatfoot [4,5]. MN is characterized by continuous common digital plantar nerve (CDPN) pain; in fact, this condition is known as “neuroma”. However, the “lesion” has perineural fibrosis, in which it does not have any pre-neoplastic or neoplastic condition [6]. The MN pathology presents with a broad spectrum of symptoms, including paresthesia, metatarsalgia and stabbing pain in the metatarsal plantar region [7]. The symptoms could also involve the nearby toes and other parts of the foot [4]. This clinical condition adversely affects the social aspect of normal life, as well as gait, shoe use and upright posture. Although specific positive tests for MN are lacking, tenderness associated with touch remains clinical evidence of the presence of Morton’s neuroma. However, the support to clinicians deriving from imaging investigations is essential for both the discovery and localization of MN [8]. So far, the diagnostic confirmation of MN’s presence is based on two different types of imaging assessments, as well as magnetic resonance imaging (MRI) and ultrasound (US). Both methodologies are able to find the target and avoid any false negative response, when the size of the neuroma is extended over 5 mm [9]. However, when identifying MN, its anatomical correspondence has always been associated with the point of maximum pain.

Non-surgical interventions for MN are a recommended treatment option before surgery [10]. However, at the moment, there is no clarity about which treatment option is the most effective. Essentially, it is possible to divide clinical approaches with curative intent into two main lists: the first includes all conservative procedures (CPs), while the second includes unconservative procedures (UPs). Biz et al. [4] recently described a detailed list of techniques used for the management of MN. The list of CPs certainly includes corticosteroid injection, radiofrequency ablation (RFA), fascial manipulation [7], laser therapy and alcohol injection [4]. Most of them were carried out with the support of US-guided (USG) procedure [11,12]. Mainly alcoholization and corticosteroid therapy are often performed via direct USG injection [13,14,15,16]. Up-front surgery is indicated when clinicians evaluate the absence of benefits derived from CPs. The list of UPs includes neurectomy [17,18] (i.e., the enucleation of CDPN) and selective neurolysis [19,20].

Surgical treatment can be performed with two different approaches: dorsal or plantar. Both appear to have no negative impact on the clinical outcome of the MN patient, while appearing to only impact the aesthetic effect [18,21]. Alcoholization and corticosteroids are widely considered conservative procedures; however, they are associated with a large list of potential side effects, including skin necrosis, digital ischemia [4], skin depigmentation [22] and plantar fat and [4] pad atrophy [23].

**Figure 1:**
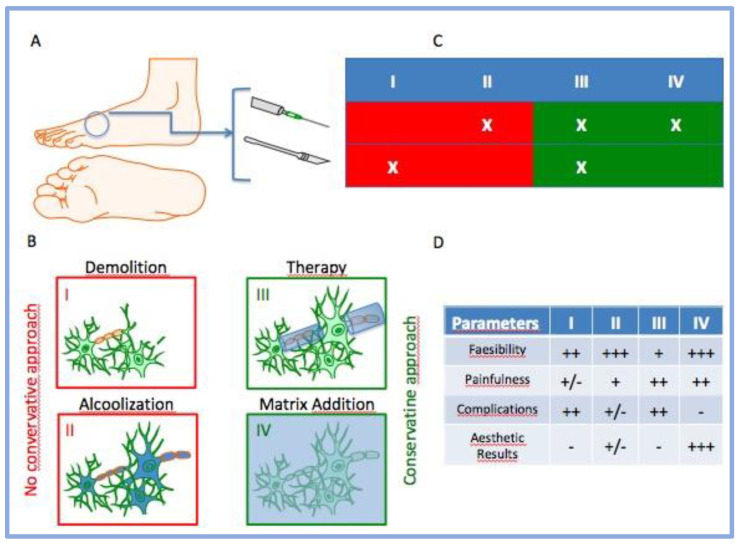
Simplification of clinical approach against MN and their cognate behaviors. Legend. (**A**) Representation of typical clinical procedure (injection and surgery); (**B**) the emerging four principal treatments of MN; (**C**) table represents the possible combinations between the procedure and treatment; (**D**) score of principal behaviors for some clinical aspects after MN treatments (− = negative; +/− = partially positive; + = positive; ++ = very positive; +++ = extremely positive).

There are several conservative proposals in the literature, but none can be considered the gold standard [24]. The use of injectable medical devices based on collagen use is another procedure (not mentioned above), which may provide a promising alternative to MN treatment [25]. This approach has good tissue compatibility, low immunogenicity and sufficient mechanical strength to support tissue regeneration. Collagen also appears to provide a guideline for regenerated bioactivity that can effectively enhance regeneration [26,27,28]. Given the absence of previous experience with collagen-based GUNA medical devices in the field of MN treatment, based on its properties and safety in use, the purpose of this study report was to test the efficacy (in terms of the functional and clinical results of a collagen-based treatment) on patients who had long-standing complaints of CDPN compressive neuropathy. Furthermore, looking at the figure below, we also intended to summarize the main differences between infiltrative-based procedures and surgery in the clinical management of MN (Figure 1).

## 2. Materials and Methods

### 2.1. Patients

Five consecutive patients (Table 1) were admitted to the Rehabilitation Medicine and Neurorehabilitation outpatient service at ASST Niguarda’s hospital (Piazza dell’Ospedale maggiore, 3; Milan, Italy). None of the patients reported either systemic disorders or previous traumas or surgical interventions to the foot, nor allergies or intolerances, nor body weight changes or the wearing of specific footwear.

**Figure 2:**
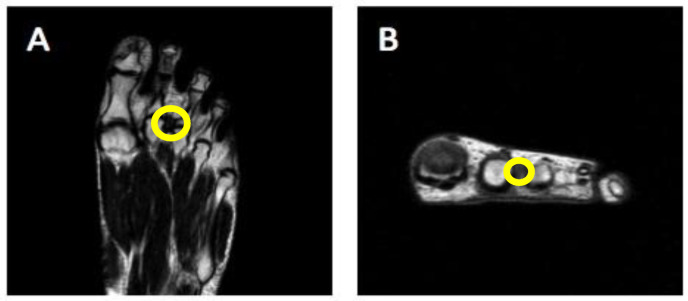
(**A**,**B**) MR imaging of Morton’s neuroma, identifiable as a hypointense soft-tissue nodule at the level of the second intermetatarsal space. The presence of MN is indicated by a yellow circle.

Indeed, the five enrolled patients had not undergone previous infiltrative or surgical treatments, had not undergone physical therapies in the last 3 months and did not change their footwear during the time-frame observation period. All of them had already treated the foot pain limiting weight-bearing activities. The patients reported a variable degree of pain during walking, paresthesia in the toes and pain when subjected to axial compression. Based on medical history and physical examination, compressive neuropathy of the common plantar digital nerve was suspected in all patients. As a whole, considering anamnestic information, all five subjects were classified as Morton’s neuroma (MN) patients (Table 2) through magnetic resonance imaging (MRI) (Figure 2) and/or ultrasound (US) analyses.

### 2.2. Injection Procedure

**Figure 3:**
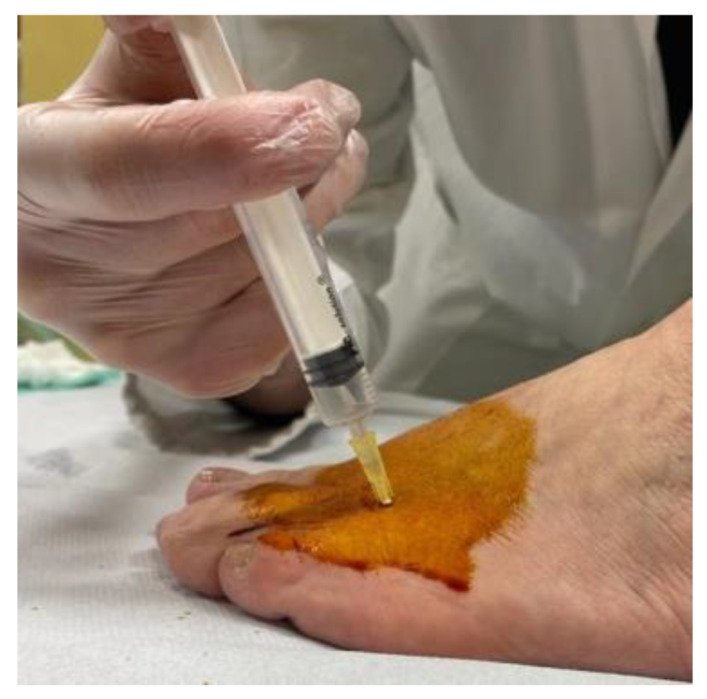
Representation of injection of porcine type I collagen added with 2% lidocaine hydrochloride, after target identification via ultrasound guidance.

We decided to treat the patients with a series of three injections of 2 mL of type I porcine collagen added with 2% lidocaine hydrochloride at weekly intervals, using an indirect US-guided (IUSG) approach. After a full and clear description of the study, the patient was invited to sign informed consent. Injections were performed by a single doctor with years of experience in ultrasound-guided infiltrative treatment. Each patient was confined to a bed with his knee flexed and his foot on the bed. The injection site was identified by using the US at the II or III interdigital space on the dorsal face of the foot; then, a 26G (13 mm) needle was inserted into the location identified, and the collagen was slowly injected. All the injections were performed at the point of maximum pain (Figure 3).

### 2.3. Porcine Type 1 Collagen

The porcine type I collagen was obtained in the following formulation: a single vial with a 2 mL final volume containing porcine type I collagen, Colocynthis, NaCl and sterile water. The product is classified as a medical device (CE; Class III), available as MD-NEURAL.

MD-NEURAL (GUNA spa, Milan, Italy) is a medical device designed to reduce neuropathic and muscular associated pain, counteracting physiological and pathological joint deterioration. Previous clinical evidence has been reported on both the efficacy and safety of MD-NEURAL in cases of the following neuropathic accuracies, neuralgia and associated neuropathic pain [26,27,28].

### 2.4. Evaluation Criteria after Treatment and Follow-Up of Patients

Patients’ timelines were established during the inclusion in the present study. All patients received three injections at a weekly interval, following a recent protocol [29]. The clinical evaluations were performed at the time of enrolment (T0); before the second injection (T1); before the third injection (T2); and one month (T3), three months (T4) and six months (T5) after the last injection. Scores on both the AOFAS scale and VNS for pain were harvested for all patients.

**Figure 4:**
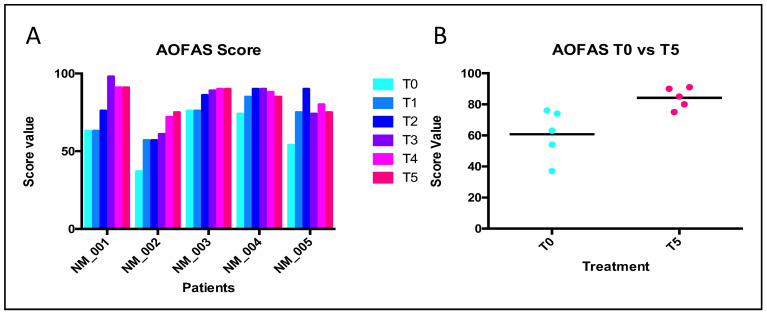
Results of AOFAS questionnaire. Legend: (**A**) ANOVA analyses. Source of variation: patients = 45.23, *p* < 0.0001; time of treatment = 36.52, *p* = 0.003; (**B**) paired *t*-test analyses. *p* = 0.0089.

**Figure 5:**
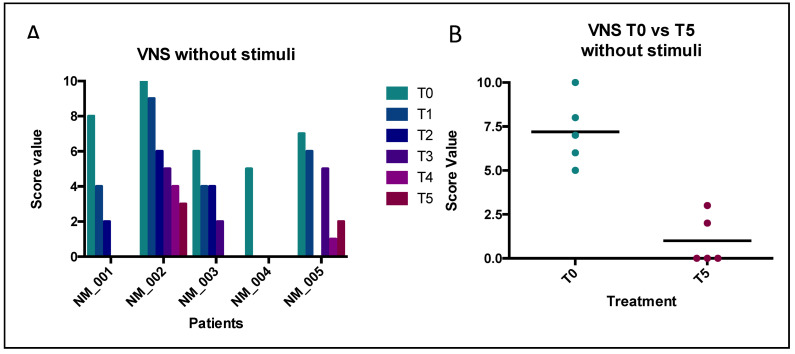
Results of VNS questionnaire (patients at rest). Legend: (**A**) ANOVA analyses. Source of variation: patients = 33.81, *p* < 0.0001; time of treatment = 52.53, *p* < 0.0001; (**B**) paired *t*-test analyses. *p* = 0.0004.

**Figure 6:**
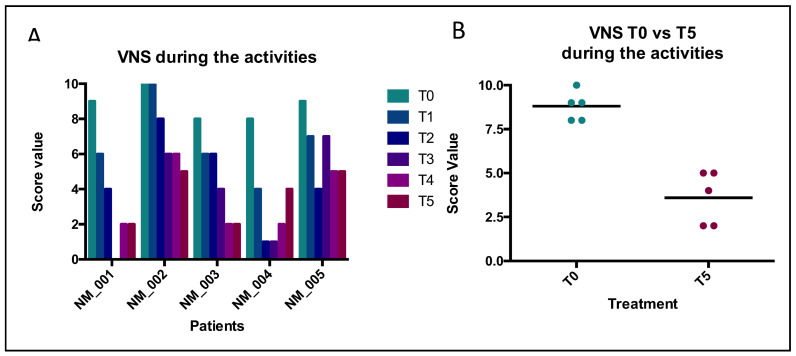
Results of VNS for self-reported pain (during weight-bearing activities). Legend: (**A**) ANOVA analyses. Source of variation: patients = 30.99, *p* = 0.0003; time of treatment = 51.55, *p* < 0.0001; (**B**) paired *t*-test analyses. *p* = 0.0009.

The AOFAS score ranges from 0 to 100 points; the best results are associated with higher scores. The VNS was used to assess pain both at rest and during weight-bearing activities. It ranges from 0 to 10; 0 represents the absence of pain, while 10 represents the maximum describable pain. The trends of collected values are illustrated in the following Figure 4, Figure 5 and Figure 6. The patients were fully compliant; during the procedures, no one of them ever referenced being in pain, and no adverse events were observed during the follow-up management.

### 2.5. Statistical Analyses:

In order to evaluate the significant statistical differences among all MN patients and their cognate treatments, ANOVA analyses (using Dunnett’s multiple comparers test) were performed. In addition to assessing the effectiveness of treatment for all patients, the paired *t*-test was also performed. We acquired significant statistical data according to the *p* < 0.05.

## 3. Results

### 3.1. Follow-Up

The follow-up was positively closed in all patients. The three scores were assessed for each patient at all the times established above in the Section 2. In total, 90 data were censored.

### 3.2. AOFAS Score

According to the criteria established above, the results of the AOFAS questionnaire are reported in Figure 4. The trends were positive for all patients, and all of them obtained a significant final benefit (Figure 4). The AOFAS mean values progressively increased, starting from T0, with a mean value of 60.80 (CI 95% 40.940–80.660) to the T5 point, in which the mean value was equal to 83.20 (CI 95% 75.810–92.590; *p* < 0.0001). The *t*-test also revealed a significant statistical difference by comparing the T0 vs. T5 treatment (*p* = 0.0089). In particular, the treatment protocol seemed to have an effect of stratification on the patients, in terms of the SD between T0 and T5 (15.99 and 6.760, respectively).

### 3.3. VNS Score

Regarding the analysis of the VNS ratings, the results were divided into two different lists, with the first reporting the quantification of pain at rest, while the second included the quantification of pain during weight-bearing activities. In both cases, the analyses revealed a positive trend for the two evaluation groups.

#### 3.3.1. VNS Score without Stimuli

Nonetheless, the VNS showed a similar trend observed for the AOFAS questionnaire, in which it is mandatory to remark that the positive effect of treatment is associated with a depletion in the score. Here, we obtained the same significant final benefit (Figure 5). The mean values of the VNS score without stimulation progressively decreased, starting from T0, with a mean value of 7.20 (CI 95% 4.812–9.588) to T5, with a mean value of 1.00 (CI 95% −0.756–2.756; *p* < 0.0001). Indeed, the *t*-test also revealed a significant statistical difference, comparing the T0 vs. T5 treatment (*p* = 0.0004). In this case, the results suggest a homogeneous positive effect, because the SDs between T0 and T5 were very close to each other (1.924 and 1.414, respectively).

#### 3.3.2. VNS Score after Activities

Nevertheless, the VNS scale regarding the data acquired during the stimulation after treatment showed a significant final benefit for all patients (Figure 6). Looking for the VNS results, the mean values progressively decreased, starting from T0, with a mean value of 8.86 (CI 95% 7.761–9.839) to T5, with a mean value of 3.60 (CI 95% 1.717–5.483; *p* < 0.0001). Indeed, the *t*-test also revealed a significant statistical difference, comparing the T0 vs. T5 treatment (*p* = 0.0009). In this case, the results do not suggest a homogeneous positive effect or the stratification of the patients, because the SD between T0 and T5 seemed to be different (0.836 and 1.517, respectively). However, the Delta VNS (VNS/T0–VNS/T5), showed a mean value equal to 5.20 points.

## 4. Discussion

Non-operative treatment is the first approach to Morton’s neuroma; surgical options should be considered when conservative treatments do not have enough of an effect [24]. There are various proposals in the literature, in some cases with controversial evidence of effectiveness: the limitation of weight-bearing activities, the use of shoes with low heels and metatarsal padding, orthotic insoles, oral non-steroidal drugs and extracorporeal shock wave therapy. Infiltrative therapy can be a valid therapeutic solution; it involves the use of corticosteroids, alcohol and phenol [4], botulinum [13], toxin or capsaicin [16]. Among them, corticosteroid injection was used most frequently as the most accessible and effective conservative treatment modality for patients with Morton’s neuroma, with improvements in outcome measures at 12 months [16,23]. However, it must be considered that local side effects of these injections have been reported in some studies, including skin atrophy, skin depigmentation and atrophy of the subcutaneous fat pad [4,22,23]. Furthermore, 1 year after the steroid injection, one-third of patients may still require surgical removal due to the recurrence of pain [23].

In this framework, we treated our patients with type I collagen through indirect ultrasound-guided injections (IUSGIs) [30,31]. Our results demonstrated that all patients received a large benefit after treatment with porcine type I collagen, thanks to its advantages such as high biocompatibility, the ability to facilitate reductions in pain and the ability to improve mobility. The foot pain and functional limitation of our patients progressively improved and in some cases almost completely disappeared at the last follow-up. Analyses of the AOFAS and VNS (without stimuli and after activities) survey demonstrated statistically significant results in all patients after treatment. Our results showed a similar trend in comparison with another study, performing a similar trend of evaluations [29]. The same modalities of analyses were used previously to analyze the outcome after corticosteroid treatment [12,15]. Indeed, their conditions improved from the first injection and progressively improved until the end of the follow-up (Figure 4, Figure 5 and Figure 6). Furthermore, by comparing collected values at T0 vs. T5, curative attempts appear to stratify patients, reducing differences among all, according to the AOFAS questionnaire (Figure 4). The clinical benefit on pain symptoms for all patients seems to be associated with both phases: at rest and during weight-bearing activities (Figure 5 and Figure 6). These positive results could be associated with collagen application, because it is the main structural protein of multiple hard and soft tissues in the human body and plays a key role in maintaining the biological and structural integrity of the extracellular matrix (ECM), providing physical support to tissues. It offers low immunogenicity, a porous structure [27], good permeability, biocompatibility and biodegradability [28]. In addition, it is possible to hypothesize that the positive action of collagen on fibrosis can also activate positive feedback in the inflammatory process. GUNA Medical Devices based on collagen have been used with promising results in many painful and degenerative diseases of the musculoskeletal system, with no side effects ever described [25]. The Porcine Extracellular Matrix also finds a positive application in surgery by entubulating exposed nerve ends following neurectomy [32]. Although this study reported only one patient treated, the clinical evidence could open the possibility of considering surgery, not just a PU for MN treatment, as it involves the application of collagen (Figure 1). Nonetheless, the surgical positioning of porcine small intestine submucosa seems to prevent neuroma and associated pain in preclinical model [33].

Previous preclinical studies demonstrated that collagen type I compounds could induce an anabolic phenotype in tenocytes by stimulating tenocyte proliferation and migration and COL-I synthesis [34], maturation and secretion, thereby promoting tendon repair [35]. In these articles, the authors demonstrated the efficacy of collagen in acting on the homeostasis of extracellular matrix remodeling by regulating the expression of the TIMP-1 gene. In fact, we can consider MMPs genes as pro-inflammatory molecules; therefore, the physiological triage, including the fibrosis, collagen application and gene expression of the TIMP-1 gene, could explain the presence of a positive effect on the inflammatory molecular process. On the other hand, the mechanism of action of steroid injections for the treatment of MN is unclear, since the neuroma is degenerative in nature. The steroid is likely to reduce the inflammation surrounding the neuroma by reducing pain and consequently local pressure effects; however, it is reasonable consider the steroid-based treatment a palliative approach [15,23]. The alcoholization of MN in CP is well regarded, but the percentage of unconvincing side effects could open it up to criticism from clinicians [4,22].

Conversely, the structural and mechanical effects provided by the collagen and biological matrix could function as an effective natural scaffold to support cell growth, while also providing a framework for cell–cell interactions by redirecting reinnervation [34]/reorganization [35]. The in situ collagen medical device, applied via indirect ultrasound guidance, has been shown to be a safe and effective approach in improving pain and function in a patient with symptomatic Morton’s neuroma. At the same time, procedure IUSGI could constitute a good alternative approach in consideration of the simplicity, time consumption and safety of execution compared to direct USG injection (DUSGI), reducing the procedure execution time, possible infectious adverse events and time-consuming patient management (IUSGI vs. DUSGI) [30,31]. Furthermore, the reduced physiological space associated with the MN becomes a safe and easily accessible point for the IUSGI procedure in order to correctly position the collagen in the target site.

## 5. Conclusions

The present study should recapitulate the list of principal take-home messages:

- The infiltrative use of porcine type I collagen could be contemplated as a promising non-surgical therapy against MN less than 10 mm in size.
- The procedure is safe and easy to perform.
- The combined indirect US-guided injection (IUSGI) approach can allow a more precise subministration of medical devices, where the point of application fits perfectly with the point of maximum pain.
- The proposed approach places itself exactly between the conservative approach and nerve-ablative treatments, as well up-front surgery and alcohol injection.
- Collagen treatment could play an important pivotal role in the positive modulation of the inflammatory process as well as steroid treatments but avoid their side effects (probably) physically acting on the fibrosis process.

The use of collagen-based medical devices seems to have not been described before in a mini-series of MN patients. Indeed, the positive clinical and functional results and the absence of side effects allow us to propose collagen injections as an option for the treatment of MN. Future studies, especially if validated by larger cohorts of subjects and longer follow-ups, will need to confirm these findings.

## Figures and Tables

**Table 1:** Anagraphic data of MN patients (M = Male; F = Female).

ID_Code	Sex	Age (yo)
MN_001	F	61
MN_002	F	62
MN_003	F	51
MN_004	F	55
MN_005	F	44

**Table 2:** Clinical data of MN patients.

ID_Code	Imaging	Site	MN Dimension
MN_001	MRI/US	SIS	<10 mm
MN_002	US	SIS	<10 mm
MN_003	MRI/US	SIS	<10 mm
MN_004	US	SIS	<10 mm
MN_005	US	SIS	<10 mm

Legend: MRI—magnetic resonance imaging; US—ultrasound; SIS—second interdigital space. The measures were in agreement according to the comparison with MRI.

## Data Availability

Data is contained withing the article.
